# Assessment of size ordered recruitment

**DOI:** 10.3389/fnhum.2014.00532

**Published:** 2014-07-28

**Authors:** Parveen N. S. Bawa, Kelvin E. Jones, Richard B. Stein

**Affiliations:** ^1^Department of Biomedical Physiology and Kinesiology, Simon Fraser UniversityBurnaby, BC, Canada; ^2^Faculty of Physical Education and Recreation, University of AlbertaEdmonton, AB, Canada; ^3^Department of Physiology, University of AlbertaEdmonton, AB, Canada

**Keywords:** motor unit recruitment, human experimentation, size principle, muscle contraction, movement

The spinal motoneurons innervating a limb muscle are heterogeneous: they vary in diameter of cell bodies, axons and surface area of the dendritic trees, electrophysiological differences (e.g., input resistance, afterhyperpolarization, spike threshold) and contractile properties of the associated muscle units. Considering the range of motoneuron and corresponding muscle unit properties, the obvious question arose about how and which motoneurons were selected by the central nervous system for various types of contractions and movements. Elwood Henneman and his coworkers proposed an unequivocal simple pattern in a series of papers starting in 1957 from experiments conducted on reduced cat preparations (Henneman, 1957; Henneman et al., 1974). The *Size Principle* of motoneuron recruitment stated that for any net excitatory input to the motoneuron pool, motoneurons were recruited in an orderly fashion, always from small to large. Stein and coworkers demonstrated that the size principle generalized to voluntary isometric contractions in humans, which they called *Orderly Recruitment* (Milner-Brown et al., 1973). The literature on this topic is well summarized in a recent review by Heckman and Enoka (2012).

Since the publication of Henneman's seminal paper in 1957, publications on this topic have steadily accumulated. The purpose of this short article is to clarify the basic essence of size ranked or orderly recruitment of motoneurons by addressing conclusions about the alternative: selective recruitment. This requires stretching the strict boundaries imposed by some authors in understanding the *Size Principle*. For example, the anatomically defined motoneuron pools may not always coincide with the group of motoneurons being excited for a particular task; the precision of rank-ordered recruitment suggested from reduced animal preparations (Henneman et al., 1974) may not be that precise in a noisy physiological system (Stein et al., 2005). Here we elaborate on some of the factors that may have lead to the conclusions of selective or random recruitment.

Though the size principle was stated for a motoneuron pool, the initial observations made by Henneman and coworkers were not restricted to one pool. They recorded from ventral roots while stretching all triceps surae muscles; the recorded activity in individual ventral roots could have its origin in any one of the synergistic pools. One recorded action potential could be from soleus and the second from gastrocnemius. Yet orderly recruitment was observed between the collection of all motoneurons that were excited by the input, specifically the total motoneuron population of soleus, medial and lateral gastrocnemius (LG) muscles. Likewise, an anatomically defined motoneuron pool may under certain conditions be compartmentalized into task groups (Riek and Bawa, 1992) and orderly recruitment is observed within each task group. The existence of task groups does not imply size independent selective recruitment of motor unit types. Wakeling (2009) has reported recruitment of different compartments of triceps muscles in goats and humans depending on the mechanics of the movement. Selective recruitment of different compartments is akin to recruitment of different task groups; again, this observation does not imply selective recruitment if excitatory input is restricted to a subset of motoneurons. This same line of reasoning applies to the condition of fast paw shake in the cat (Smith et al., 1980). In these experiments EMG activity was observed in the *fast* LG muscle but not in the *slow* soleus muscle during the paw shake. This observation is often cited as evidence for selective recruitment of motor unit types. However, there was no discussion as to whether the motor units within the LG muscle, which is composed of type I and II muscle fibers, were recruited according to size. A subsequent study that included EMG sampling from histochemically regionalized muscles showed high activation of all muscle regions during paw shaking, which is not consistent with preferential recruitment of muscle regions rich in type II fibers during this condition (Chanaud et al., 1991). We suggest that when discussing recruitment order, the motoneuron pool should be operationally defined as the group of motoneurons that receive excitatory synaptic input to drive the functional movement, not the pool of motoneurons defined by anatomy. The validity of the *Size Principle* should then be evaluated within this operationally defined motoneuron pool to determine if recruitment proceeds from small to large.

What do orderly recruitment and selective recruitment really mean? The accepted narrative is that orderly recruitment of motor units from small to large twitch force, results in a more precise control of force and movement; this precision is more important for small and mid range forces. By maintaining the same order of recruitment, the central nervous system minimizes the computational load across a wide range of desired outputs (Henneman et al., 1974). A range of quantitative theoretical studies support this qualitative description. For example, Senn et al. (1997) used an information theoretical approach to demonstrate that orderly recruitment maximizes information content of motoneuron output that in turn minimizes the error in muscle force generation. Selective recruitment, on the other hand, refers to the hypothesis that under certain conditions the central nervous system selects motor units to enhance the force and rate of force output irrespective of the rank order of the motor unit within a motoneuron pool. To achieve this goal, selective recruitment may use preferential inhibition of small motor units. The most commonly proposed examples of selective recruitment include ballistic contractions, lengthening contractions and the preferential recruitment of fast motor units during cutaneous stimulation. However, empirical evidence from a number of laboratories failed to support the hypothesis of selective recruitment in these conditions (reviewed by Heckman and Enoka, 2012). Electrical stimulation of some pathways could produce inhibition of small motor units and excitation of larger motor units. Yet none of the behavioral studies demonstrated selective recruitment of large units with inhibition of the small ones. A possible basis for this discrepancy comes from Kernell and Hultborn (1990). They proposed that the selective excitatory and inhibitory synaptic inputs change the gain of the input-output curve of the motoneuron pool. To increase the recruitment gain, the small motoneurons are biased with inhibitory currents. Alternatively, or concurrently, the large motoneurons can be biased with excitatory currents. The opposite synaptic bias scheme can be used to decrease the gain. All motoneurons receiving excitatory input to drive the final movement, despite underlying bias inputs, should be considered part of a functionally defined pool of motoneurons. In the high gain situation, there will be a higher likelihood of random departures from strict recruitment as a result of noise—however, the general principle of rank ordered recruitment will remain.

Another factor that has contributed to a misunderstanding of the orderly recruitment is the expectation for precise rank ordered recruitment (Henneman et al., 1974). The degree of precision suggested from reduced animal preparations is not expected to hold for a noisy physiological system (Stein et al., 2005). It is important to remember that rank-ordered recruitment was originally defined for movement under healthy conditions. However, in conditions such as ageing, reinnervation, pain and fatigue, when precision decreases, other processes may obscure rank-ordered recruitment. With ageing, some motoneurons die leaving behind orphaned muscle fibers. The surviving motoneurons will sprout new terminals to reinnervate some of the orphaned fibers, thus changing the size of the muscle units (Chan et al., 2001; Gordon et al., 2004). Under these conditions, the order of recruitment will be less orderly which will lead to a decline in precision of motor output. During prolonged contractions in healthy adults, it has been shown that motoneurons fatigue and rotation of activity occurs among motor units (Manning et al., 2010). If rotation occurs among almost similar sized motor units, precision will remain unaffected. However, rotation among motor units of quite different sizes would increase the noise in motor output. Another example of disrupted rank ordered recruitment is the activity of motor units during pain (Tucker et al., 2009). These examples are not evidence for selective recruitment, but rather a decrease in precision of rank-ordered recruitment. In our experience the situation in which common sense reasoning favors selective recruitment is movements with maximum velocity over a short period of time: a ballistic movement. The reasoning is that slow motor units impede ballistic movements while exclusive recruitment of fast units would be optimal. However a recent study demonstrated that the recruitment of slow motor units does not pose any such problem (Holt et al., 2014).

The ultimate meaning of recruitment order lies in the force output of the entire musculoskeletal system for the purpose of behaviorally relevant movements. Clearly in *laboratory* conditions with simple muscles and a limited set of contractions, recruitment is orderly in terms of force (Milner-Brown et al., 1973; Calancie and Bawa, 1985; Zajac and Faden, 1985; Riek and Bawa, 1992; Jones et al., 1993). In biomechanically complex musculotendon-skeletal systems, accurately measuring the size of a motor unit may be difficult (Clamann and Schelhorn, 1988; Bodine-Fowler et al., 1990; Roy et al., 1995). The challenge for the field is to measure size and recruitment order, over relevant ranges of force during ecologically valid behavior (Jones et al., 1994). Until this is done, the question remains whether the laboratory defined *size principle of orderly recruitment* will generalize to movements of everyday life.

## Conflict of interest statement

The authors declare that the research was conducted in the absence of any commercial or financial relationships that could be construed as a potential conflict of interest.

## References

[B1] Bodine-Fowler S. Garfinkel A. Roy R. R. Edgerton V. R. (1990). Spatial distribution of muscle fibers within the territory of a motor unit. Muscle Nerve 13, 1133–1145 10.1002/mus.880131208 1702521

[B2] Calancie B. Bawa P. (1985). Voluntary and reflexive recruitment of flexor carpi radialis motor units in humans. J. Neurophysiol. 53, 1194–1200 3998806 10.1152/jn.1985.53.5.1194

[B3] Chan K. M. Doherty T. J. Brown W. F. (2001). Contractile properties of human motor units in health, aging, and disease. Muscle Nerve 24, 1113–1133 10.1002/mus.1123 11494264

[B4] Chanaud C. M. Pratt C. A. Loeb G. E. (1991). Functionally complex muscles of the cat hindlimb. V. The roles of histochemical fiber-type regionalization and mechanical heterogeneity in differential muscle activation. Exp. Brain Res. 85, 300–315 10.1007/BF00229408 1832646

[B5] Clamann H. P. Schelhorn T. B. (1988). Nonlinear force addition of newly recruited motor units in the cat hindlimb. Muscle Nerve 11, 1079–1089 10.1002/mus.880111012 3185603

[B6] Gordon T. Thomas C. K. Munson J. B. Stein R. B. (2004). The resilience of the size principle in the organization of motor unit properties in normal and reinnervated adult skeletal muscles. Can. J. Physiol. Pharmacol. 82, 645–661 10.1139/y04-081 15523522

[B7] Heckman C. J. Enoka R. M. (2012). Motor unit. Compr. Physiol. 2, 2629–2682 10.1002/cphy.c100087 23720261

[B8] Henneman E. (1957). Relation between size of neurons and their susceptibility to discharge. Science 126, 1345–1347 10.1126/science.126.3287.1345 13495469

[B9] Henneman E. Clamann H. P. Gillies J. D. Skinner R. D. (1974). Rank order of motoneurons within a pool: law of combination. J. Neurophysiol. 37, 1338–1349 4436704 10.1152/jn.1974.37.6.1338

[B10] Holt N. C. Wakeling J. M. Biewener A. A. (2014). The effect of fast and slow motor unit activation on whole-muscle mechanical performace: the size principle may not pose a mechanical paradox. Proc. R. Soc. B Biol. Sci. 281:20140002 10.1098/rspb.2014.0002 24695429 PMC3996609

[B11] Jones K. E. Bawa P. McMillan A. S. (1993). Recruitment of motor units in human flexor carpi ulnaris. Brain Res. 602, 354–356 10.1016/0006-8993(93)90702-O 8448677

[B12] Jones K. E. Lyons M. Bawa P. Lemon R. N. (1994). Recruitment of motoneurons during various behavioural tasks. Exp. Brain Res. 100, 503–508 10.1007/BF02738409 7813686

[B13] Kernell D. Hultborn H. (1990). Synaptic effects on recruitment gain: a mechanism of importance for the input-output relations of motoneuron pools? Brain Res. 507, 176–179 10.1016/0006-8993(90)90542-J 2302576

[B14] Manning C. D. Miller T. A. Burnham M. L. Murnaghan C. D. Calancie B. Bawa P. (2010). Recovery of human motoneurons during rotation. Exp. Brain Res. 204, 139–144 10.1007/s00221-010-2295-2 20490783

[B15] Milner-Brown H. S. Stein R. B. Yemm R. (1973). The orderly recruitment oh human motor units during voluntary isometric contractions. J. Physiol. 230, 359–370 4350770 10.1113/jphysiol.1973.sp010192 PMC1350367

[B16] Riek S. Bawa P. (1992). Recruitment of motor units in human forearm extensors. J. Neurophysiol. 68, 100–108 1517816 10.1152/jn.1992.68.1.100

[B17] Roy R. R. Garfinkel A. Ounjian M. Payne J. Hirahara A. Hsu E. (1995). Three-dimensional structure of cat tibialis anterior motor units. Muscle Nerve 18, 1187–1195 10.1002/mus.880181015 7659113

[B18] Senn W. K. Wyler K. Clamann H. P. Kleinle J. Lüscher H.-R. Müller L. (1997). Size principle and information theory. Biol. Cybern. 76, 11–22 10.1007/s004220050317 9050202

[B19] Smith J. L. Betts B. Edgerton V. R. Zernicke R. F. (1980). Rapid ankle extensions during paw shakes: selective recruitment of fast ankle extensors. J. Neurophysiol. 43, 612–620 7373352 10.1152/jn.1980.43.3.612

[B20] Stein R. B. Gossen E. R. Jones K. E. (2005). Neuronal variability: noise or part of the signal? Nat. Rev. Neurosci. 6, 389–397 10.1038/nrn1668 15861181

[B21] Tucker K. Butler J. Graven-Nielsen T. Riek S. Hodges P. (2009). Motor unit recruitment strategies are altered during deep-tissue pain. J. Neurosci. 29, 10820–10826 10.1523/JNEUROSCI.5211-08.2009 19726639 PMC6665526

[B22] Wakeling J. M. (2009). The recruitment of different compartments within a muscle depends on the mechanics of the movement. Biol. Lett. 5, 30–34 10.1098/rsbl.2008.0459 18842570 PMC2657740

[B23] Zajac F. E. Faden J. S. (1985). Relationship among recruitment order, axonal conduction velocity, and muscle-unit properties of type-identified motor units in cat plantaris muscle. J. Neurophysiol. 53, 1303–1322 2987433 10.1152/jn.1985.53.5.1303

